# *Operando* structural science of functional materials

**DOI:** 10.1107/S2052252521008393

**Published:** 2021-09-01

**Authors:** C. Richard A. Catlow

**Affiliations:** a Department of Chemistry, University College London, 20 Gordon Street, London, WC1H 0AJ, United Kingdom; b School of Chemistry, Cardiff University, Park Place, Cardiff, CF10 3AT, United Kingdom

**Keywords:** *operando* structural science, functional materials, editorial

## Abstract

This editorial gives a brief overview of *operando* structural science of functional materials with emphasis on catalytic science.

Classical structural science examined samples using diffraction and/or spectroscopic techniques under ambient conditions. Studies as a function of temperature have, of course, become routine and high-pressure experiments also have made a major contribution to structural science. In recent years, however, the availability of more sophisticated sample environments has enabled a rapid growth in the use of ‘*in situ and operando*’ techniques, in which a functional material, such as a catalyst, is probed under conditions which resemble as closely as possible those used under the real operating environment, with measurements often being made with time resolution. The field is growing rapidly in sophistication and poses exciting challenges to, and opportunities for, the structural science of materials.

A terminological confusion can arise from the use of both ‘*in situ*’ and ‘*operando*’, with the latter denoting an experiment where the structural measurements are made together with measurements relating to its functional performance, which, for example, for a catalytic material would be the reaction product yield and distribution. A good illustration of an *operando* structural study was reported recently in **IUCrJ** by Rabøl Jørgensen *et al.* (2020 ▸), who investigated the thermoelectric material Zn_4_Sb_3_ using a set-up which permitted simultaneous measurements of X-ray diffraction data and electrical resistance on samples subject to an electric current. From the analysis of the data, they were able to infer that zinc ions are mobile and also to demonstrate sample degradation at higher current densities. Their work paves the way for further *in operando* studies of thermoelectrics – materials of growing importance in energy technologies – and, as the authors comment, of solid-state battery and piezo- and ferroelectric materials.

The most extensive applications of *in situ/operando* techniques are probably those in catalytic science, using synchrotron-based X-ray diffraction and X-ray absorption spectroscopy. Here they have had a huge impact in recent years and good reviews of earlier work in this area are available from Beale *et al.* (2010 ▸), Newton & van Beek (2010 ▸) and Bentrup (2010 ▸). Several recent examples can be found in the *Faraday Discussions* held this year on *Reaction Mechanisms in Catalysis*, including the study of Bugaev *et al.* (2021 ▸) which examined the industrially relevant reaction of ethyl­ene hydrogenation using palladium catalysts, monitored by both XRD and XAS, illustrating the well established and increasing trend to apply multiple measurement techniques during *in situ* experiments. The data obtained allow the structural evolution of the working catalyst to be monitored as well as the transitions between metallic, hydride and carbide phases of palladium as the catalytic reaction progresses. Another excellent illustration of the current state of the art is provided by the recent study of van Ravenhorst *et al.* (2021 ▸) on the topical Fisher–Tropsch Co/TiO_2_ catalyst (which catalyses the synthesis of hydro­carbons from syngas, *i.e.* CO/H_2_ feed). They again combine synchrotron-based XAS with both synchrotron- and laboratory-based XRD to study the evolution of the catalyst on stream for 48 h. They are able to follow the formation of cobalt carbide as the reaction progresses, although the product distribution is largely unaffected. Interestingly, the formation of carbide is detected in XAS before XRD, as the former is more sensitive to short-range order. The paper nicely illustrates how detailed structural information on the evolution of complex catalytic systems can be obtained by this type of multi-technique *operando* study. A further recent example showing technical innovation is provided by the work of Matras *et al.* (2021 ▸), who used advanced tomographic imaging techniques in an *operando* study of a Ni–Pd/CeO_2_–ZrO_2_/Al_2_O_3_ catalyst during the partial oxidation of methane. The study gives detailed information both on the evolution of the metal and oxide species during the catalytic reaction and on the role of the heterogeneity in the catalyst particles.

A significant recent development is the ability to undertake experiments within a catalytic reactor, which is nicely illustrated by the work of Nieuwelink *et al.* (2021 ▸), again from the recent *Faraday Discussions*. The study followed hydrogenation reactions in a microreactor and was able to probe single particles during the reaction. ‘In reactor’ experiments can also give spatially resolved information as in the recent work of Decarolis *et al.* (2021 ▸) who investigated the widely studied selective catalytic oxidation of ammonia using a catalyst comprising palladium supported on alumina. The study shows that the nature of the catalytic species changes along the reactor and that these changes can be correlated with product distribution. This type of spatial analysis will grow in importance as the ability to undertake spatially resolved experiments develops.

*In operando* methodologies are not confined to X-ray techniques; they have been exploited, albeit less widely, using neutron scattering. Good examples in catalytic science include the work using neutron spectroscopy which was reported by Parker (2011 ▸), who clarified the role of surface hydroxyls in CO oxidation over a model palladium catalyst; the study also illustrates the power of neutron spectroscopic techniques to probe catalytic systems, which would be difficult to study by photon-based spectroscopies. A further illustration is provided by Youngs *et al.* (2013 ▸) who employed neutron total scattering methods to probe time-resolved catalytic chemistry in a Pt/SiO_2_ catalyzed benzene hydrogenation reaction.

Finally, we should note that although the extensive range of applications in catalysis have had notable impact, *operando* techniques can be applied to many other classes of functional material, such as energy materials, highlighted earlier. Structural studies of functional materials will increasingly be made under ‘*operando*’ conditions. **IUCrJ** would welcome submissions in this exciting and growing field.
